# *Staphylococcus aureus* delta toxin modulates both extracellular membrane vesicle biogenesis and amyloid formation

**DOI:** 10.1128/mbio.01748-23

**Published:** 2023-10-05

**Authors:** Xiaogang Wang, Divakara SSM Uppu, Seth W. Dickey, Dylan J. Burgin, Michael Otto, Jean C. Lee

**Affiliations:** 1 Division of Infectious Diseases, Department of Medicine, Brigham and Women's Hospital and Harvard Medical School, Boston, Massachusetts, USA; 2 Pathogen Molecular Genetics Section, Laboratory of Bacteriology, National Institute of Allergy and Infectious Diseases, Bethesda, Maryland, USA; 3 Department of Veterinary Medicine, Virginia-Maryland Regional College of Veterinary Medicine,University of Maryland, Bethesda, Maryland, USA; St. Jude Children's Research Hospital, Memphis, Tennessee, USA

**Keywords:** *Staphylococcus aureus*, extracellular membrane vesicles, phenol-soluble modulins, delta-toxin, amyloid fibrils

## Abstract

*Staphylococcus aureus* secretes phenol-soluble modulins (PSMs), a family of small, amphipathic, secreted peptides with multiple biologic activities. Community-acquired *S. aureus* strains produce high levels of PSMs in planktonic cultures, and PSM alpha peptides have been shown to augment the release of extracellular membrane vesicles (MVs). We observed that amyloids, aggregates of proteins characterized by a fibrillar morphology and stained with specific dyes, co-purified with MVs harvested from cell-free culture supernatants of community-acquired *S. aureus* strains. δ-toxin was a major component of amyloid fibrils that co-purified with strain LAC MVs, and δ-toxin promoted the production of MVs and amyloid fibrils in a dose-dependent manner. To determine whether MVs and amyloid fibrils were generated under *in vivo* conditions, we inoculated mice with *S. aureus* harvested from planktonic cultures. Bacterial MVs were isolated and purified from lavage fluids recovered from infected animals. Although δ-toxin was the most abundant PSM in lavage fluids, amyloid fibrils could not be detected in these samples. Our findings expand our understanding of amyloid fibril formation in *S. aureus* cultures, reveal the important roles of δ-toxin in amyloid fibril formation and MV biogenesis, and demonstrate that MVs are generated *in vivo* in a staphylococcal infection model.

**IMPORTANCE:**

Extracellular membrane vesicles (MVs) produced by *Staphylococcus aureus* in planktonic cultures encapsulate a diverse cargo of bacterial proteins, nucleic acids, and glycopolymers that are protected from destruction by external factors. δ-toxin, a member of the phenol soluble modulin family, was shown to be critical for MV biogenesis. Amyloid fibrils co-purified with MVs generated by virulent, community-acquired *S. aureus* strains, and fibril formation was dependent on expression of the *S. aureus* δ-toxin gene (*hld*). Mass spectrometry data confirmed that the amyloid fibrils were comprised of δ-toxin. Although *S. aureus* MVs were produced *in vivo* in a localized murine infection model, amyloid fibrils were not observed in the *in vivo* setting. Our findings provide critical insights into staphylococcal factors involved in MV biogenesis and amyloid formation.

## INTRODUCTION

*Staphylococcus aureus* is a primary cause of invasive infections in humans, including bacteremia, endocarditis, pneumonia, and wound infections, leading to morbidity, mortality, and excessive healthcare costs (1). By employing a diverse array of surface-associated and secreted virulence factors, *S. aureus* colonizes and invades host tissues and evades the host immune response (2 – 7). Lacking secretion systems (T3SS, T4SS, and T6SS) that transport Gram-negative bacterial virulence factors directly into host cells (8), *S. aureus* secretes its exoproteins to the external environment (8 – 11), where they may be inactivated by neutralizing antibodies or enzymes with proteolytic or hydrolytic activities. However, *S. aureus* also generates extracellular membrane vesicles (MVs), and virulence determinants packaged within MVs (12 – 17) are protected from destruction by external factors.

MVs are spherical membrane nanoparticles released by prokaryotes, eukaryotes, and archaea (18). The *S. aureus* MV cargo includes cytosolic, surface, and membrane proteins, as well as nucleic acids, glycopolymers, and secreted proteins, such as pore-forming toxins, superantigens, and proteases (12 – 17). Purified MVs are cytolytic to multiple cell types (12, 14, 15, 19), induce atopic dermatitis-like inflammation in mice (19, 20), and elicit the production of pro-inflammatory mediators (16, 19 – 23). MV production is enhanced by environmental stresses typically encountered by *S. aureus* during infection (24 – 26). Although many *S. aureus* strains produce MVs *in vitro* (12 – 15, 17, 23, 27), the generation of MVs *in vivo* during staphylococcal infection has not yet been documented.

Although the biogenesis of MVs generated by Gram-positive bacteria remains poorly understood, phenol-soluble modulins (PSMs) have been shown to be critical for MV formation in *S. aureus*. PSMs, produced by multiple staphylococcal species, are genome-encoded, amphipathic peptides with alpha-helical structures (28, 29). They are classified into two types: the α-type peptides with 21–26 amino acids (PSMα1, α2, α3, α4, and δ-toxin) and the β-type peptides with 44 amino acids (PSMβ1 and β2) (30). PSMα1–4 peptides enhance the release of MVs by altering the cell membrane due to their surfactant-like characteristics, thus increasing membrane fluidity and promoting MV biogenesis (14, 31). Under appropriate conditions, various PSM peptides have been shown to form amyloid fibrils alone or in combination (32 – 36). Most of the latter studies were performed *in vitro* with synthetic PSM peptides or under biofilm growth conditions.

In this report, we investigated the roles of PSMα peptides, PSMβ peptides, and δ-toxin in the generation of *S. aureus* MVs and evaluated the relationship between PSM production and amyloid formation under planktonic culture conditions. Both PSMα peptides and δ-toxin enhanced *S. aureus* MV production, and δ-toxin formed amyloid fibrils that co-purified with MVs harvested from culture supernatants. MVs were produced *in vivo* in an *S. aureus* air pouch infection model, but amyloid fibrils could not be detected in lavage fluids. Our study provides fundamental insights into the role of *S. aureus* PSM peptides in MV biogenesis and amyloid fibril formation in planktonic cultures.

## RESULTS

### Strain-dependent production of MVs and amyloid fibrils in planktonic *S. aureus* cultures

**Fig 1:**
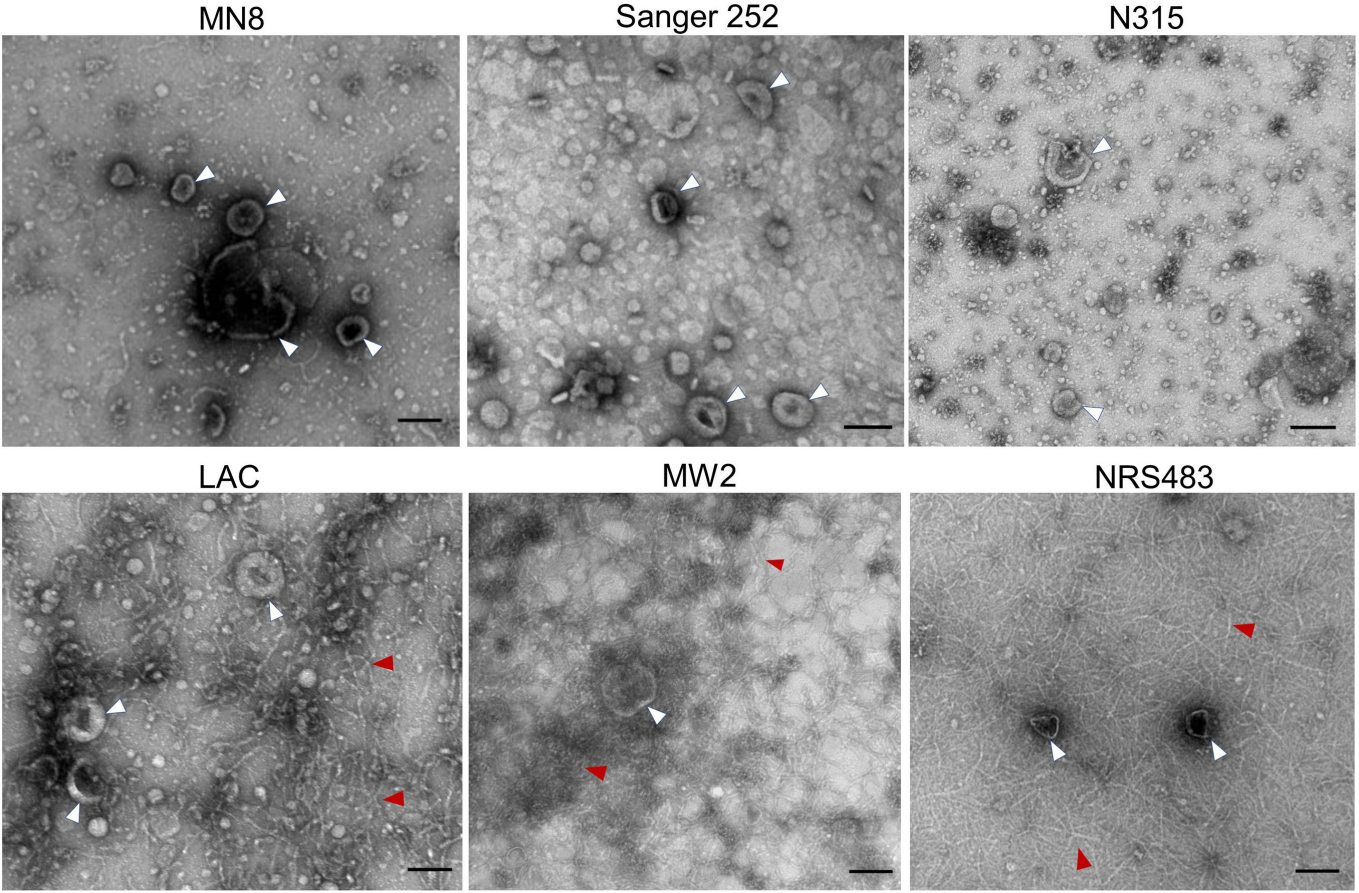
Electron micrographs of MVs purified from the post-exponential cultures of indicated *S. aureus* strains. MN8, Sanger 252, and N315 are hospital-acquired *S. aureus* strains, whereas strains LAC, MW2, and NRS483 are community-acquired isolates. MVs and fibrils in the images are marked with white and red arrowheads, respectively. Scale bars, 100 nM. Images are representative of three independent experiments.

We purified MVs from culture supernatants of hospital-acquired *S. aureus* isolates (ST5 strain N315, ST36 strain Sanger 252, and ST30 strain MN8), as well as the more virulent community-acquired *S. aureus* isolates (ST8 strain LAC, ST1 strain MW2, and ST59 strain NRS483). Negatively stained samples imaged by transmission electron microscopy (TEM) revealed diverse MV morphologies (Fig. 1). MVs generated by strains MN8, Sanger 252, and N315 varied somewhat in their appearance, consistent with previous reports (37). However, MV preparations purified from community-acquired *S. aureus* strains MW2, ST59, and LAC were distinctive as they contained an abundance of amyloid-like fibrils (Fig. 1).

Despite employing a combination of tangential flow filtration, ultracentrifugation, density-gradient ultracentrifugation, and diafiltration to achieve MV purification, fibrils co-purified with MV preparations from the prototype *S. aureus* USA300 strain LAC. We considered that fibrils visualized by TEM could be an artifact of our MV purification process. To address this, we prepared additional samples wherein we omitted the steps of tangential flow filtration, Opti-prep gradient ultracentrifugation, and diafiltration. Abundant fibrils were observed by TEM in the crude LAC MV pellet (Fig. S1A), but not in the crude Sanger 252 sample (Fig. S1B). To determine whether the observed fibrils were amyloid in nature, purified MV samples from each strain were stained with the amyloid-specific dye thioflavin T (ThT) (38). LAC MVs displayed a dose-dependent increase in fluorescence that was lacking in MVs prepared from Sanger 252 (Fig. S1C). These data demonstrate that amyloid fibrils were formed in planktonic cultures of community-acquired *S. aureus* strains, and that the fibrils co-purified with MVs generated *in vitro*.

### δ-toxin plays a dominant role in amyloid formation in *S. aureus* planktonic cultures

Because synthetic PSMs or PSMs produced under biofilm conditions formed amyloid fibrils *in vitro* (32 – 36, 39, 40), we hypothesized that PSMs contributed to amyloid fibril formation in planktonic cultures of *S. aureus* LAC. The expression of PSMs is growth phase-dependent, and *agr* dysfunctional mutants lack detectable PSM production (28). Accordingly, MVs harvested from exponential phase cultures of strain LAC were not associated with fibrils (Fig. S2A). Likewise, MVs purified from post-exponential cultures of JE2∆*agr* (Fig. S2B) were free of fibrils. In contrast, MV samples purified from post-exponential cultures of the pore-forming toxin mutant LAC∆*lukAB*∆*hlgACB*∆*lukED*∆*pvl*∆*hla* (41) (Fig. S2C) or JE2∆*atl* (Fig. S2D) showed abundant fibrils, indicating that neither cytolysins nor cytoplasmic proteins released by the major autolysin Atl modulate fibril formation (42).

**Fig 2:**
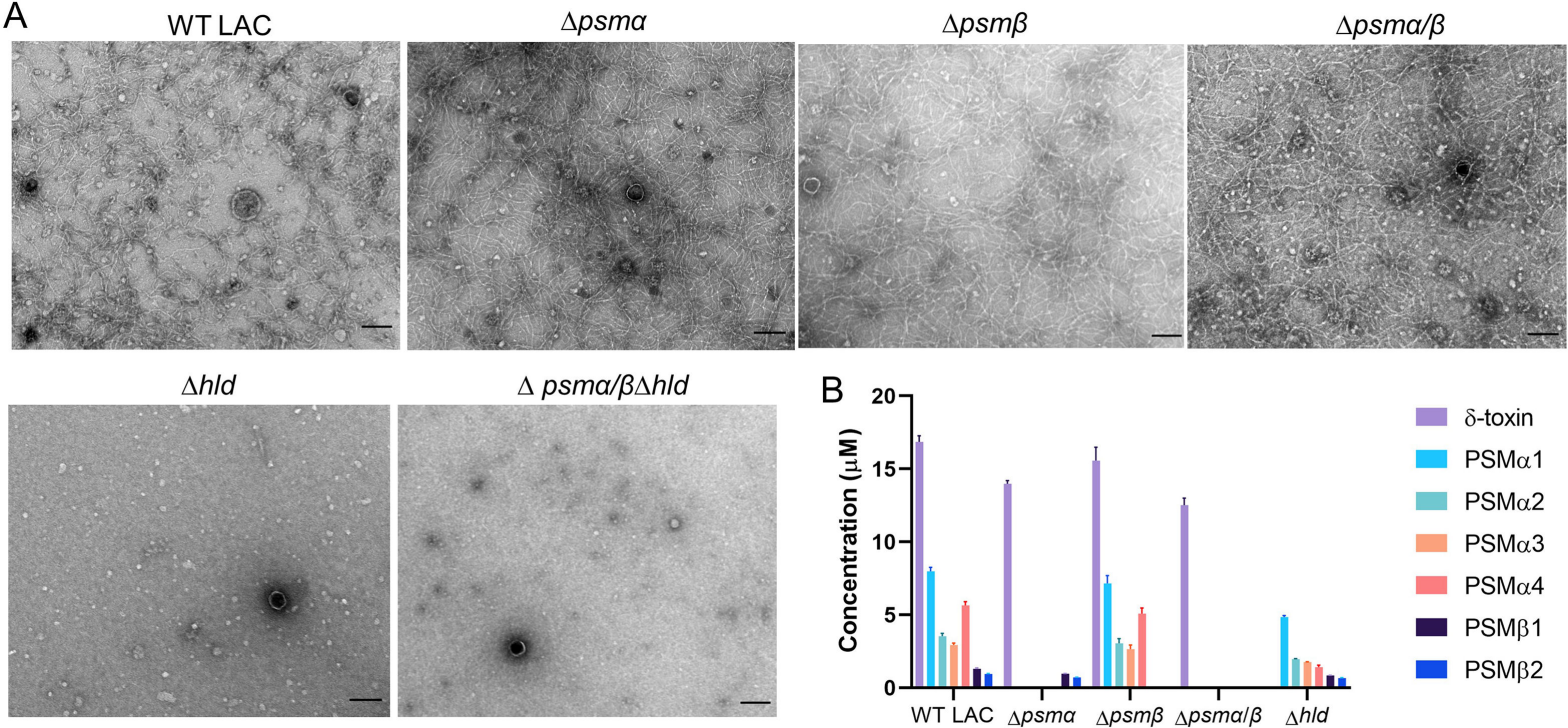
Effects of PSMs on amyloid fibril formation in post-exponential *S. aureus* LAC cultures. (**A**) MVs purified from WT LAC or indicated *psm* mutants were negatively stained and imaged by TEM. The experiments were performed at least two times with different batches of MV samples, and a representative image for each sample is shown. Scale bar, 100 nm. (**B**) The concentrations of individual PSM peptides in culture supernatants of strain LAC and its isogenic PSM mutants. Values shown are means + SEM.

To investigate whether PSMs play a role in amyloid fibril formation, we purified MVs from the wild-type (WT) strain LAC and LAC PSM mutants. Fibrils were present in MV samples purified from post-exponential cultures of LAC, LAC∆*psmα*, LAC∆*psmβ*, and LAC∆*psmα/β* (Fig. 2A). In contrast, fibrils were absent from samples lacking δ-toxin (LAC∆*hld* and LAC∆*psmα/β*∆*hld*). Likewise, when crude MVs were pelleted by ultracentrifugation from filter-sterilized culture supernatants of strains LAC (Fig. S3A) or MW2 (Fig. S3B), fibrils were observed in TEM images of MVs from the parental strains and the ∆*psmα*, ∆*psmβ,* and ∆*psmα/β* mutants, but fibrils were absent in images of the ∆*hld* and ∆*psmα/β/*∆*hld* MVs. When we eliminated the ultracentrifugation step and concentrated *S. aureus* supernatants by ultrafiltration only, fibrils were observed by TEM in supernatants from WT LAC but not from the ∆*hld* mutant (Fig. S4A).

The PSM concentrations in 8-h culture supernatants of strain LAC and its isogenic PSM mutants are shown in Fig. 2B. As expected, δ-toxin was the most abundant PSM peptide detected. The δ-toxin mutant displayed decreased levels of PSMα1, PSMα2, PSMα3, and PSMα4 in the culture supernatant compared to the WT strain, a finding consistent with a previous report (43) describing the association of PSMα peptides with the *S. aureus* cell surface. This binding of PSMα to the bacterial surface was partially inhibited by δ-toxin, resulting in an increase in the amount of bacterial cell surface-associated PSMα peptides and a decrease in PSMα peptides in the culture supernatant of a δ-toxin mutant (43).

**Fig 3:**
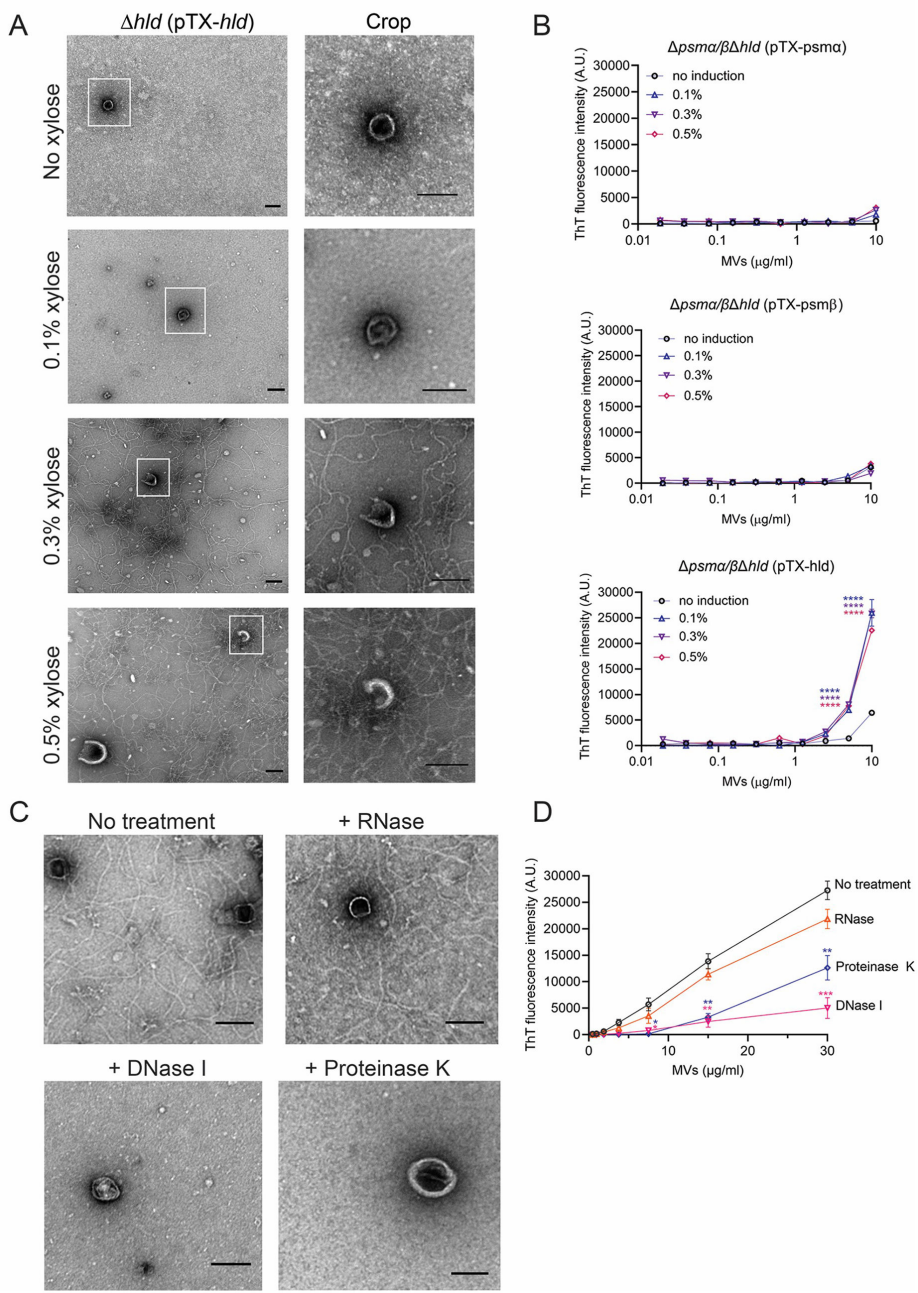
The formation of amyloid fibrils in post-exponential cultures of *psm* mutant strains complemented with *psmα, psmβ,* or *hld*. (**A**) Electron micrographs of MV samples purified from cultures of LAC∆*hld* expressing *hld* under the control of a xylose-inducible promoter. A cropped and magnified field from each image is shown. (**B**) ThT fluorescence analyses of MV samples purified from the ∆*psmα/β*∆*hld* mutant that was complemented with various *psm* genes and induced with increasing concentrations of xylose. (**C**) Electron micrographs of MV samples purified from *S. aureus* LAC cultivated in the presence or absence of DNase I, RNase, or proteinase K. MVs from the samples were negatively stained and imaged by TEM. Scale bars, 100 nM. (**D**) Fluorescence of ThT-stained MV samples purified from treated or untreated LAC cultures. ThT fluorescence was expressed as the mean ± SEM (*n* = 3). Data were compared to the uninduced or untreated sample by the Student *t* test. test. **P  <  0.01, ***P  <  0.001, ****P < 0.0001.

We complemented LAC∆*hld* with pTX*-hld* (44), which allowed us to control the expression of *hld* by induction with 0%, 0.1%, 0.3%, or 0.5% xylose. When the cultures reached the post-exponential growth phase, the MVs from each culture were purified and imaged by TEM. Fibrils were observed in MV samples purified from cultures with *hld* induction by xylose concentrations ≥0.3% (Fig. 3A).

To confirm that δ-toxin was solely responsible for amyloid fibril formation, the LAC∆*psm*α*/β*∆*hld* mutant was complemented with *psm*α*1–4*, *psmβ1–2,* or *hld* using inducible vector pTX (44). MVs from each culture were stained with ThT. Samples from the ∆*psm*α*/β*∆*hld* (pTX-*hld*) strain induced with 0.1%, 0.3%, or 0.5% xylose exhibited significant fluorescence at MV concentrations ≥5 µg/mL (Fig. 3B). In contrast, MVs purified from the ∆*psm*α*/β*∆*hld* (pTX-*psm*α) or ∆*psm*α*/β*∆*hld* (pTX-*psmβ*) cultures exhibited no appreciable fluorescence in the presence or absence of xylose. The pTX plasmids did not induce identical concentrations of the corresponding peptides after induction with 0.5% xylose. Although PSMα peptides were induced to the highest extent (2.13 ± 0.32 µM), no fibrils were seen in these samples. δ-toxin levels reached 1.05 ± 0.05 µM, whereas the concentration of the PSM*β* peptides was the lowest at 0.63 ± 0.10 µM. These data demonstrate that only δ-toxin promoted amyloid fibril formation in *S. aureus* planktonic cultures.

Because *S. aureus* extracellular DNA (eDNA) promotes the formation of amyloid fibrils under biofilm conditions (45), we cultivated strain LAC in the presence or absence of DNase I or RNase. Separate cultures were grown in the presence of proteinase K to digest extracellular proteins, including δ-toxin. MVs were harvested by ultracentrifugation and visualized by TEM. Whereas fibrils were present in MV samples from untreated *S. aureus* cultures or those treated with RNase, the fibrillar content was reduced when the bacteria were cultivated in the presence of DNase or proteinase K (Fig. 3C). Likewise, ThT fluorescence of MVs purified from cultures incubated with DNase or proteinase K was markedly reduced compared to that of WT MVs (Fig. 3D). Because both enzymes prevented fibril formation during bacterial growth, our results suggest that δ-toxin and eDNA modulate amyloid fibril formation in *S. aureus* planktonic cultures.

### δ-toxin is a major structural component of amyloid fibrils

**Fig 4:**
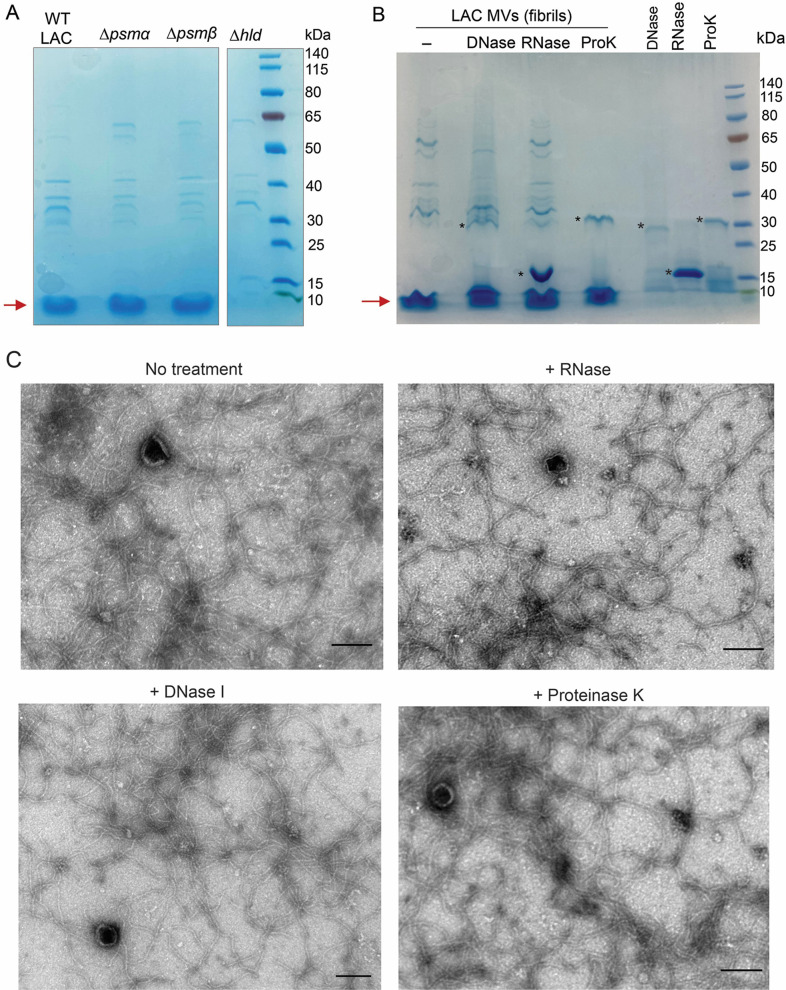
Analysis of enzyme-treated and untreated *S. aureus* MV samples. (**A**) SDS-PAGE of MVs purified from post-exponential cultures of WT LAC and its PSM mutants or (**B**) purified LAC MV samples treated with DNase, RNase, or proteinase K (ProK). A band with a molecular weight <10 kDa is indicated by a red arrow. *, indicates major bands associated with enzymes used for MV treatments. (**C**) Electron micrographs of purified LAC MVs before or after treatment with ProK, DNase I, or RNase. Scale bar, 100 nM.

Because fibrils were associated with MVs purified from WT LAC, ∆*psm*α, and ∆*psmβ*, but not with MVs purified from LAC∆*hld*, we examined whether there were differences in their MV protein profiles as assessed by SDS-PAGE. Untreated MV-associated fibrils did not enter the gel, but samples boiled with SDS and a reducing reagent disaggregated the fibrils prior to sample loading. Four to six major protein bands (~30 to 80 kDa) were observed in MV samples from LAC and its PSM mutants (Fig. 4A). Of note, a protein band with a molecular mass <10 kDa was observed in MVs purified from WT LAC, ∆*psm*α, and ∆*psmβ* but not from LAC∆*hld*, suggesting that this band was δ-toxin. Purified MV samples were untreated or digested with DNase, RNase, or proteinase K before analysis by SDS-PAGE. As shown in Fig. 4B, DNase or RNase treatment did not markedly alter the protein profile of the MV samples. In contrast, the 30–80 kDa bands were degraded by proteinase K, whereas the <10 kDa band resisted degradation. The <10 kDa protein band was excised and subjected to LC-MS/MS, and δ-toxin represented >99.9% of its components (Table S1). When purified MVs were treated for 1 h with proteinase K, DNase I, or RNase before TEM imaging, amyloid fibrils remained in both treated and untreated samples (Fig. 4C). Our results indicate that DNase I and proteinase K prevent fibril formation during bacterial growth, but that once mature fibrils are formed, they resist degradation by both enzymes.

### *S. aureus* PSMα peptides and δ-toxin promote MV production

**Fig 5:**
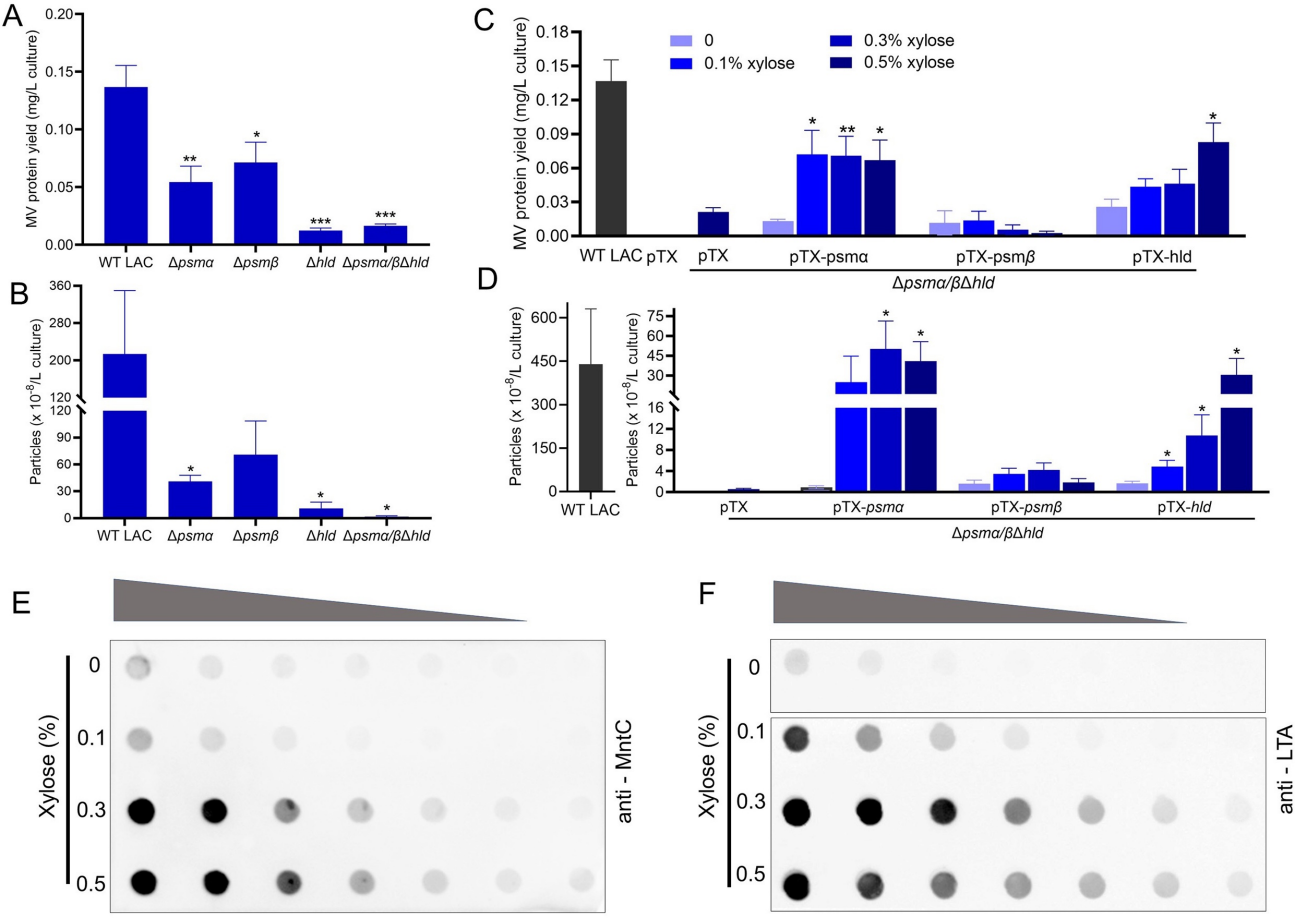
The effect of PSM peptides on the yield of *S. aureus* MVs. Production of MVs from WT LAC and its isogenic mutants lacking *psmα*, *psmβ*, or *hld* was quantified by (**A**) total MV protein abundance or (**B**) MV particle numbers assessed by nanoparticle tracking analysis (*n* = 3). MV production from cultures of the LAC∆*psmα/β*∆*hld* mutant complemented with *psmα, psmβ*, or *hld* genes induced with indicated concentrations of xylose was quantified by (**C**) total MV protein yield or (**D**) MV particle numbers (*n* = 3–5). Twofold serial dilutions of MV samples purified from ∆*psmα/β*∆*hld* (pTX-*hld*) cultures induced by indicated concentrations of xylose were evaluated by dot blots probed with anti-MntC sera (**E**) or anti-lipoteichoic acid (LTA) antibodies (**F**). The dot immunoblot assay was performed at least two times with similar results; a representative blot is shown. MV protein yield and MV particle quantification experiments were expressed as mean + SEM. The data were analyzed using one-way ANOVA with Dunnett’s multiple comparison test; *P  <  0.05, **P  <  0.01, ***P  <  0.001.

Mutation of the LAC *psmα* genes significantly reduced MV protein yield (Fig. 5A) and particle numbers (Fig. 5B) to a greater extent than mutation of the *psmβ* genes. The Δ*hld* mutant showed the lowest MV protein yield and particle numbers, equivalent to that of the ∆*psm*α*/β*∆*hld* mutant. We extended these findings by complementing the ∆*psm*α*/β*∆*hld* mutant with genes encoding either PSMα peptides, PSMβ peptides, or δ-toxin. Gene expression in the complemented mutants was induced with 0%, 0.1%, 0.3%, or 0.5% xylose. As shown in Fig. 5C and D, the ∆*psm*α*/β*∆*hld* mutant carrying either the empty vector pTX or pTX-*psmβ* showed minimal MV yields. In contrast, abundant MV production occurred in the ∆*psm*α*/β*∆*hld* (pTX-*psm*α) cultures when the *psm*α*1–4* genes were induced by 0.1–0.5% xylose. MV yields and particle numbers from induced cultures were significantly higher than those of uninduced cultures. Induction of *hld* in ∆*psm*α*/β*∆*hld* (pTX-*hld*) cultures resulted in xylose inducible, dose-dependent increases in MV production (Fig. 5C and D). MVs from the ∆*psm*α*/β*∆*hld* mutant complemented with pTX-*psmα*, pTX-*psm*β, or pTX-*hld* and induced with 0.5% xylose were visualized by TEM (Fig. S4B).

As an alternative approach, we utilized dot immunoblots to estimate relative MV concentrations in samples of LAC∆*psm*α*/β*∆*hld* (pTX-*hld*) cultivated in increasing concentrations of xylose. A xylose dose-dependent increase in signal was observed in samples probed with antibodies to the lipoprotein MntC (Fig. 5E) or lipoteichoic acid (Fig. 5F), antigens abundant in *S. aureus* MVs (16, 26). These findings support our conclusion that induction of δ-toxin by xylose enhances MV production.

### Detection of *S. aureus* MVs in an air pouch infection model

Although many *S. aureus* isolates produce MVs *in vitro* (12 – 15, 17, 23, 27), the generation of MVs *in vivo* during staphylococcal infection is unproven. Visualization of MVs *in vivo* is difficult because these nanoparticles can only be seen by electron microscopy. Mammalian cells also secrete vesicles (exosomes and microvesicles) (46), and this represents a challenge for the purification of *S. aureus* MVs from body fluids. Synthetic PSM peptides form amyloid fibrils *in vitro*, but the formation of fibrils *in vivo* has not yet been documented.

**Fig 6:**
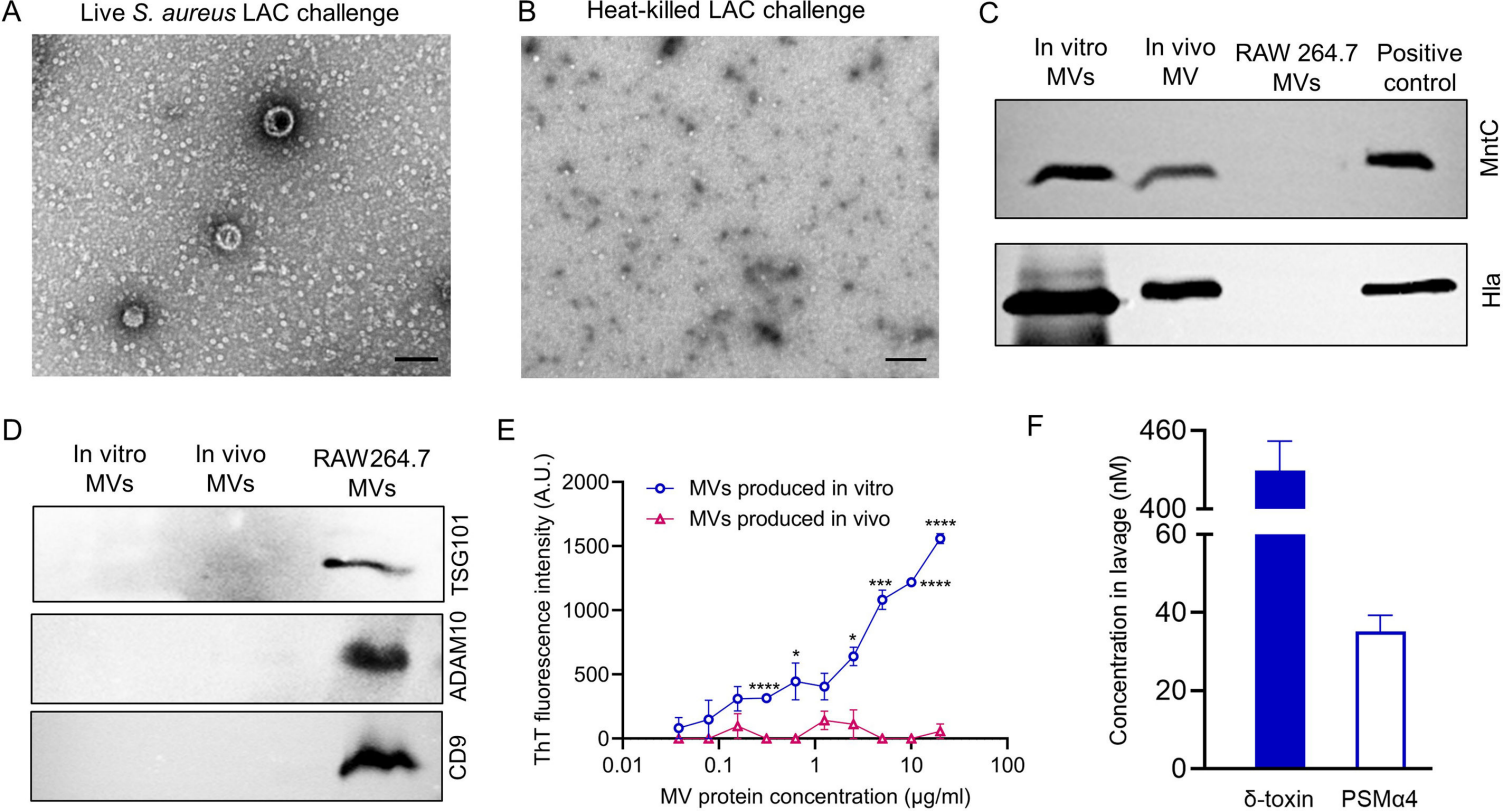
*S. aureus* MVs are generated *in vivo* in a murine air pouch infection model. Electron micrographs of MV samples purified from pouch lavage fluids of mice infected with viable (**A**) or heat-killed *S. aureus* LAC (**B**). Scale bar, 100 nm. (**C**) Immunoblots show *S. aureus* MV reactivity with antibodies to alpha toxin (Hla) and MntC. (**D**) Eukaryotic MV markers (TSG101, ADAM10, and CD9) only reacted with MVs purified from murine RAW264.7 macrophages. Data from panels (A–D) are representative of at least two independent experiments. (**E**) ThT fluorescence data were expressed as means ± SEM (*n* = 3) and analyzed by the Student *t* test. *P < 0.05, ***P < 0.001, ****P < 0.0001. (**F**) Concentrations of indicated PSM peptides in the air pouch lavage fluids.

We employed a murine air pouch infection model (Fig. S5A) to evaluate *in vivo* production of MVs. Air pouches were inoculated with live or heat-killed (HK) *S. aureus* LAC at an inoculum of ~10^8^ CFU/mouse. The mice were euthanized at 48 h, and the pouches were lavaged with 1 mL phosphate-buffered saline (PBS) and cultured quantitatively. Viable bacteria were not recovered from mice given 10^8^ CFU HK *S. aureus*. Bacterial growth was observed in ~75% of the pouches inoculated with viable *S. aureus*, and ~50% of the total pouches show a ≥2-fold increase in bacterial numbers. Samples from mice that showed bacterial replication *in vivo* were pooled and purified. MVs were visualized by TEM in samples from mice inoculated with *S. aureus* LAC (Fig. 6A) but not from mice given HK LAC (Fig. 6B), consistent with previous reports that MVs are only generated by live bacteria (47).

To assess whether MVs recovered from mouse air pouch lavage fluids were microbial in origin, the samples were analyzed by immunoblots. MVs purified from infected mice were reactive with antibodies to *S. aureus* alpha toxin and MntC (Fig. 6C), antigens associated with *S. aureus* LAC MVs generated *in vitro* (16, 26). Antibodies to the eukaryotic vesicle markers TSG101 (48), ADAM10 (49), and CD9 (48, 49) were reactive with vesicles purified from culture supernatants of murine RAW 264.7 macrophages (Fig. 6D), but not with MVs harvested from air pouch lavage fluids or from *in vitro S. aureus* cultures. We compared the lipid content of MV samples by thin-layer chromatography (TLC). Although not identical, the lipid banding pattern of *in vivo* MVs more closely resembled that of the *S. aureus* membranes and *in vitro* MVs than that of eukaryotic lipids derived from RAW 264.7 cells and vesicles (Fig. S6). Taken together, these data strongly suggest that MVs purified from air pouch infections are *S. aureus* in origin.

Amyloid fibrils were not observed in crude MVs directly pelleted from pouch lavage fluids (Fig. S5B) or in purified “*in vivo*” MV samples (Fig. 6A). To confirm this observation, MVs harvested either from *in vitro* cultures or infected air pouches were serially diluted and stained with ThT. MV samples purified from *in vitro* cultures showed a dose-dependent increase in ThT fluorescence, whereas ThT fluorescence was not measurable in MV samples purified from infected mice (Fig. 6E). To determine whether δ-toxin was present in the lavage fluids from infected mice, we analyzed pooled samples by RP-HPLC. Only δ-toxin and PSMα4 were detected in the lavage fluids ( Fig. 6F).

## DISCUSSION

As a family of small amphipathic, surfactant-like toxins, PSM peptides have multiple biological activities, including cytolysis (28), stimulation of host inflammatory responses, modulation of host innate and adaptive immunity (50), and effects on biofilm maturation (44). PSM monomers, especially PSMα and PSMβ peptides, have been shown to self-assemble into amyloid-like fibrils *in vitro*, and these amyloid fibrils stabilize *in vitro* biofilms (34 – 36). PSMs contribute to *in vitro* biofilm structuring and detachment, as well as *S. aureus* dissemination from *in vivo* biofilms (44, 51). Because evidence is lacking that PSM amyloid formation occurs in biofilms formed *in vivo*, whether this process is biologically relevant remains questionable.

Zhou et al. ammonium sulfate-precipitated PSMs from overnight culture supernatants of the community-acquired *S. aureus* strain MW2, and fibrils composed of δ-toxin were identified in ethanol-extracted precipitates (52). In their studies, formylated δ-toxin formed fibrils, whereas deformylated δ-toxin formed oligomer complexes with PSMα peptides. Only the formylated δ-toxin fibrils bound the amyloid-indicator dye ThT (52). Somerville et al. reported that δ-toxin accumulates in the culture medium in formylated and deformylated forms during the exponential phase of bacterial growth, whereas formylated δ-toxin accumulates during the post-exponential growth phase (53). These observations are compatible with our findings that fibrils were produced in cultures grown to the post-exponential growth phase when formylated δ-toxin levels exceeded those of the other PSMs.

Unlike data generated with synthetic PSMα1, PSMα3, PSMβ1, and PSMβ2 peptides that form amyloid fibrils *in vitro* (33, 35, 36), our data indicate that amyloid fibril formation relied on dose-dependent *hld* expression in planktonic *S. aureus* LAC cultures. δ-toxin is abundant in culture supernatants of virulent, community-acquired *S. aureus* strains, and for many strains it is the most abundant secreted protein (28, 54). Amyloid fibrils formed in post-exponential cultures of strain LAC bound ThT and were composed primarily of δ-toxin. No fibrils were detected in MVs purified from cultures of hospital-associated *S. aureus* isolates, which is consistent with the fact that these strains produce lower levels of δ-toxin *in vitro* compared to community-acquired strains (28, 55, 56).

Because bacterial culture supernatants are complex, we postulated that factors other than δ-toxin in the spent culture medium could influence PSM amyloid formation. Schwartz et al. reported that eDNA interacted with PSMα1 to promote amyloid formation in *S. aureus* biofilms, and that an ∆*atl* mutant lacking eDNA did not form fibrils (45). Our data indicate that MV samples purified from ∆*atl* planktonic cultures still contained fibrils (Fig. S2D), and thus distinct mechanisms of fibril formation occur in planktonic cultures. When we cultivated *S. aureus* LAC in the presence of DNase I or proteinase K, these enzymes inhibited fibril formation in growing cultures. *S. aureus* MVs are associated with nuclease-susceptible bacterial DNA (57), and thus our findings suggest that eDNA may interact with δ-toxin to promote amyloid fibril formation in *S. aureus* cultures. In contrast, when we treated purified MV preparations with DNase I, RNase, or proteinase K, the amyloid fibrils were resistant to degradation. This result is consistent with previous reports demonstrating that eDNA is protected from DNase-mediated degradation by its interaction with PSM peptides (40), and that amyloid fibrils are resistant to proteinase K digestion (58, 59).

Several studies have reported that PSMα peptides are essential for *S. aureus* MV biogenesis (14, 31). Although δ-toxin proved to be integral to the formation of amyloid fibrils in *S. aureus* cultures, our data indicate that, like PSMα (14), δ-toxin also plays a key role in MV biogenesis. A LACΔ*hld* mutant was equivalent to the triple PSM mutant in terms of reduced MV yield and particle numbers (Fig. 5A). WT levels of MVs were restored to the PSM-negative mutant by complementation with either *psmα1–4* or *hld* (Fig. 5C). PSMs are believed to promote MV production by targeting the cytoplasmic membrane and increasing membrane fluidity due to their surfactant-like activity. Indeed, a LACΔ*psm*α/β/*hld* triple mutant showed significantly reduced membrane fluidity compared to the WT strain, and the addition of synthetic PSMα3 or PSMα2 enhanced bacterial membrane fluidity in a dose-dependent fashion (31). An *agr* mutant lacks PSMs due to strict regulation of *psm* expression by AgrA (60), and JE2Δ*agr* showed a ~70% reduction in MV protein yield.

We employed a murine air pouch infection model to determine whether *S. aureus* MVs were generated *in vivo* and whether “*in vivo*” MVs might be associated with amyloid fibrils. The air pouch infection model offers the benefit of an *in vivo* compartment that is easy to sample, and it has been utilized to study inflammation (61, 62), bacterial pathogenesis, host responses to infection (63, 64), and the protective efficacy of multiple vaccine antigens (64). Strain LAC MVs were produced in this infection model, but whether the generation of MVs impacts the pathogenesis of staphylococcal infection remains to be determined.

We did not detect amyloid fibrils associated with MVs harvested from the murine air pouch infection model. Although δ-toxin was the most abundant PSM in lavage fluids, host-derived factors could impede fibril formation or degrade fibrils *in vivo*. Najarzadeh et al. reported that human plasma fibrinogen inhibits fibrillation of PSMα1, PSMβ1, and PSMβ2 and induces fibrillation in PSMα3, but its impact on δ-toxin fibrillation was not investigated (65). Serum lipoproteins have been shown to bind to and neutralize the biologic activities of PSMs (66).

In summary, our study investigates the relationship between amyloid formation by PSM peptides and their contribution to the generation of MVs in *S. aureus* cultures. Our work revealed that δ-toxin was responsible for fibril formation in cultures of strain LAC and that, like PSMα, was critical for MV production *in vitro*. We isolated and purified *S. aureus* MVs from body fluids of animals experimentally infected with *S. aureus*, providing evidence that MVs are produced *in vivo*. However, amyloid fibrils were not observed in association with MVs purified from infected mice. Our findings provide fundamental insights into the generation of MVs during infection to further our understanding of the contribution of MVs to staphylococcal pathogenesis. The infection model that we used to evaluate MV production *in vivo* can also be used to investigate MV production by other bacterial pathogens.

## MATERIALS AND METHODS

### Bacterial strains and growth conditions

*S. aureus* strains (listed in Table S2) were cultivated with aeration in tryptic soy broth (Difco) at 37°C to the exponential (OD_650nm_ = 0.9; 4 h), post-exponential (OD_650nm_ = 1.2; 4.5 h), or stationary (8 h) phase of growth. For induction of expression of *psm*α, *psmβ*, or *hld* in the LAC∆*psmα/β*∆*hld* mutant, the strains were cultivated at 37°C in Luria broth with 0%, 0.1%, 0.3%, or 0.5% xylose and supplemented with 12.5 µg/mL tetracycline. To complement LAC∆*hld*, pTX-*hld* was transformed into RN4220 by electroporation and then transduced to LAC∆*hld* with ϕ80α. Isolation and purification of MVs from *in vitro* cultures were described previously (14, 26) and are detailed in the Supplemental Methods.

### ThT fluorescence

To detect amyloid fibrils, 50 µL of PBS containing 0.4 mM ThT (Thermo Fisher) was mixed with an equal volume of MVs or PBS in black 96-well flat bottom plates. After a 30-min incubation at room temperature, the fluorescence of ThT (*E*_*x*_/*E*_*m*_ = 438 nM/495 nM) was measured.

### Quantification of PSM peptides

Unprocessed culture supernatants and lyophilized lavage fluid samples dissolved in 6 M guanidine hydrochloride were analyzed in triplicate by reversed-phase HPLC/MS as described (67) but with a 2.1 × 50 mm^2^ C18 column for lavage fluid analysis. PSMs were quantified using Agilent MassHunter software by summing the extracted ion chromatogram peak area from two ionized (*m/z*) species per PSM. Concentrations of PSMs in the samples were determined by calibration with standard curves of synthetic N-formylated peptides.

### Enzyme treatments

*S. aureus* was cultivated to the post-exponential phase of growth in tryptic soy broth supplemented with or without 200 µg/mL DNase I, RNase, or proteinase K. Filter-sterilized culture supernatants were ultracentrifuged at 150,000 × *g* at 4°C for 3 h. The samples were resuspended in 100 µL sterile PBS, and the presence of amyloid fibrils was analyzed by TEM and ThT fluorescence. Purified MV samples containing amyloid fibrils were incubated for 1 h at 37°C with or without 200 µg/mL DNase I, RNase, or proteinase K before examination by TEM.

### Dot immunoblot analysis of MV yield

LAC∆*psmα/β*∆*hld* (pTX-*hld*) cultures were induced with 0%, 0.1%, 0.3%, or 0.5% xylose and cultivated to the post-exponential growth phase. MVs purified from each culture were serially diluted and applied to nitrocellulose membranes using a 96-well Bio-dot apparatus (Bio-Rad). Immunodetection of MntC or LTA was performed as described previously (26).

### Purification of MVs generated *in vivo* in the murine air pouch infection model

Animal experiments were carried out in accordance with the PHS Policy on Humane Care and Use of Laboratory Animals. Our animal use protocol (2016N000429) was approved by the Brigham and Women’s Hospital Institutional Animal Care and Use Committee. Air pouches were created by the subcutaneous injection of 3 mL air into the dorsolateral region of mice on days 0 and 3. *S. aureus* strain LAC was cultivated to the logarithmic phase of growth, and the bacteria were harvested and washed to remove MVs generated *in vitro*. Air pouches were inoculated on day 5 with 0.5 mL of *S. aureus* containing ~10^8^ CFU. The mice were euthanized at 48 h, and the pouches were lavaged with 1 mL PBS. An aliquot of each air pouch lavage sample was serially diluted and plated to quantify the *S. aureus* CFU/mL. Samples that showed ≥2-fold bacterial growth *in vivo* were pooled and centrifuged to remove bacteria and host cells. To isolate and purify MVs generated *in vivo*, filter-sterilized pouch lavage fluids were subjected to ultracentrifugation (150,000 × *g*) at 4°C for 3 h. The crude MV pellet was gently suspended in PBS and further purified by “top down” OptiPrep density gradient ultracentrifugation, as outlined previously (68). Aliquots of each OptiPrep fraction were subjected to SDS-PAGE and evaluated by immunoblots with polyclonal antibodies to alpha toxin (Hla) (69) (1 µg /mL) or MntC (1:1,000 serum dilution) (26). Reactive fractions (6–9) were pooled, and the OptiPrep medium was removed by diafiltration with PBS. The samples were negatively stained and examined by TEM.

To validate whether MVs recovered from air pouches were microbial in origin, 5 µg MVs purified from *in vitro* LAC cultures or from pooled air pouch lavage fluids were subjected to SDS-PAGE and analyzed by immunoblots. Controls included recombinant His-Hla and His-MntC and eukaryotic vesicles purified from culture supernatants of murine RAW 264.7. Eukaryotic MV markers were detected by rabbit anti-TSG101 monoclonal antibody (1 µg/mL; Thermo Fisher; MA1-23296), rabbit anti-ADAM10 monoclonal antibody (1 µg/mL; Thermo Fisher; MA5-32616), and rabbit anti-CD9 monoclonal antibody (1 µg/mL; Cell Signaling; 98327S).

For TLC analysis, *S. aureus* membranes were prepared from lysostaphin-induced protoplasts as described by Wilkinson et al. (70). Lipids were extracted from purified MVs and the membranes of *S. aureus and* RAW 264.7 cells following the Bligh and Dyer protocol (71) using a solution of water/chloroform/methanol (0.9/1/1; vol/vol/vol). The samples were applied to TLC plates (Nano-SIL G UV254 ALUGRAM Nano-SIL-G; Macherey-Nagle Inc.) and resolved in a chamber saturated with chloroform/ethanol/water/triethylamine (35/35/7/35; vol/vol/vol/vol). Lipid standards included L16:0 phosphatidylglycerol and 16:0 phosphatidylserine (Avanti Polar Lipids, Inc.). The plates were stained by nebulization with 0.05% (wt/vol) primulin (Sigma) and visualized by fluorescence at 488 nm.

## Data Availability

Data supporting the findings of this manuscript are available from the corresponding author upon reasonable request.

## SUPPLEMENTAL MATERIAL

Supplemental text and figures mbio.01748-23-s0001.pdf Supplemental methods and Figures S1 to S6.

Table S1 mbio.01748-23-s0002.docx Proteins identified in the SDS-PAGE gel band of <10 kDa.

Table S2 mbio.01748-23-s0003.docx
*S. aureus* strains used in this study.

ASM does not own the copyrights to Supplemental Material that may be linked to, or accessed through, an article. The authors have granted ASM a non-exclusive, world-wide license to publish the Supplemental Material files. Please contact the corresponding author directly for reuse.
