# Supplementary material for: Staphylococcus aureus delta toxin modulates both extracellular membrane vesicle biogenesis and amyloid formation
Source: mBio. 2023 Oct 5;14(5):e01748-23. doi: 10.1128/mbio.01748-23 (PMC10653798; doi:10.1128/mbio.01748-23)
Supplement: Supplemental text and figures — Supplemental methods and Figures S1 to S6. [file mbio.01748-23-s0001.pdf]

Staphylococcus aureus Delta Toxin Modulates both Extracellular Membrane Vesicle Biogenesis and Amyloid Formation

Xiaogang Wang[a], Divakara SSM Uppu[a,*], Seth W. Dickey[b,c], Dylan J. Burgin[b], Michael Otto[b], and Jean C. Lee[a,#]

[a] Division of Infectious Diseases, Department of Medicine, Brigham and Women's Hospital and Harvard Medical School, 181 Longwood Avenue, Boston, MA USA 02115

[b] Pathogen Molecular Genetics Section, Laboratory of Bacteriology, National Institute of Allergy and Infectious Diseases, National Institutes of Health, Bethesda, MD, USA 20814

[c] Department of Veterinary Medicine, Virginia-Maryland Regional College of Veterinary Medicine, University of Maryland, College Park, MD, USA 20740

[#] To whom correspondence may be addressed. Email: jclee@bwh.harvard.edu

[*] Current address: Cytiva, 14 Walkup Dr., Westborough, MA, USA 01581

Supplemental Methods

Purification of MVs generated in vitro*.* Filter-sterilized *S. aureus* culture supernatants were concentrated 40-fold by tangential flow filtration with a 100-kDa polyether sulfone membrane system (Centramate, Pall Corp.). The concentrated supernatants were ultracentrifuged at 150,000 × g at 4°C for 3 h to pellet the crude MVs. To remove membrane fragments and protein aggregates, the pellet was gently suspended in PBS and purified by density-gradient ultracentrifugation with 40% to 15% Opti-prep medium. After centrifugation at 140,000 × g for 16 h at 4°C, aliquots of 1 ml gradient fractions were analyzed by SDS-PAGE and silver staining. Fractions with similar protein profiles were pooled, and Optiprep was removed by diafiltration with PBS. Purified MVs were filtered (0.45 µm) and stored at -80°C. MVs were visualized by TEM as described previously (1), and protein concentrations were determined with Bio-Rad protein dye. MV particle enumeration was performed using a Zetaview Nanoparticle Tracking Analyzer (Particle Metrix) with the following settings: camera sensitivity, 85.0; shutter, 70.0; frame rate, 30 frames per second; and temperature, 25°C. Analyses were performed with the in-built Zetaview software 8.02.31.

Supplemental Figures

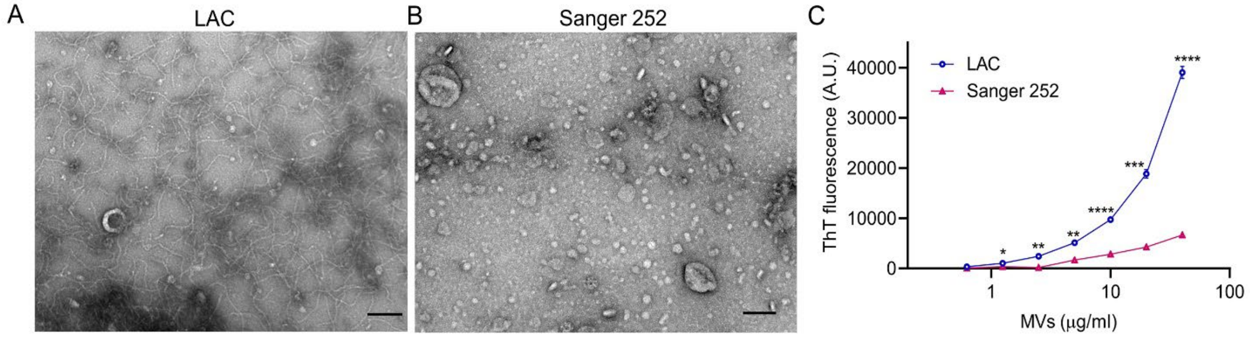

Figure S1. Comparison of MV-associated amyloid fibril formation by two *S. aureus* strains. Electron micrographs of crude MVs prepared from post-exponential cultures of (A) *S. aureus* LAC or (B) Sanger 252. Scale bar, 100 nm. (C) ThT fluorescence of MVs purified from LAC and Sanger 252 is expressed as the mean ± SEM (n=3) and analyzed using the Student *t*-test. *$P < 0.05$, **$P < 0.01$, ***$P < 0.001$, ****$P<0.0001$.

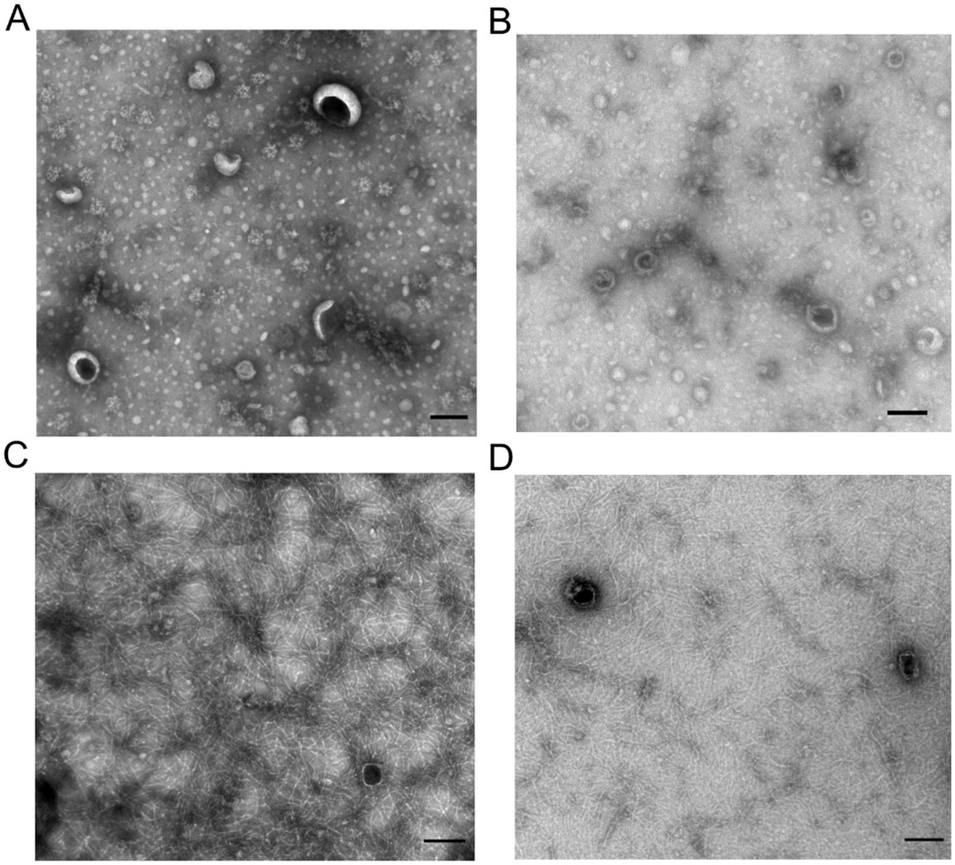

Figure S2. Electron micrographs of MV samples purified from *S. aureus* broth cultures. MVs purified from (A) strain LAC cultivated to exponential phase (4 h); (B) JE2Δ*agr* cultivated to post-exponential phase (4.5 h); (C) LACΔ*lukAB*Δ*hlgACB*Δ*lukED*Δ*pvl*Δ*hla* cultivated to post-exponential phase; and (D) JE2Δ*atl* cultivated to post-exponential phase. Scale bars, 100 nm.

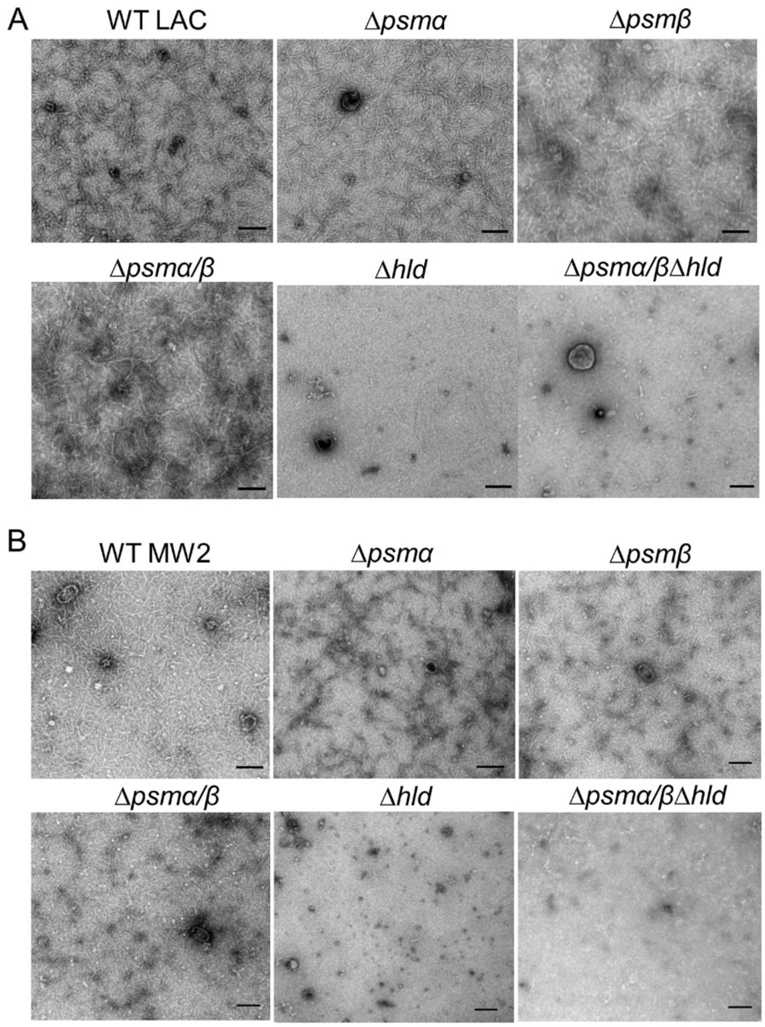

Figure S3 Electron micrographs of crude MV samples prepared from *S. aureus* LAC (A) or MW2 (B) and the indicated PSM mutants cultivated to the stationary growth phase. Scale bars, 100 nm.

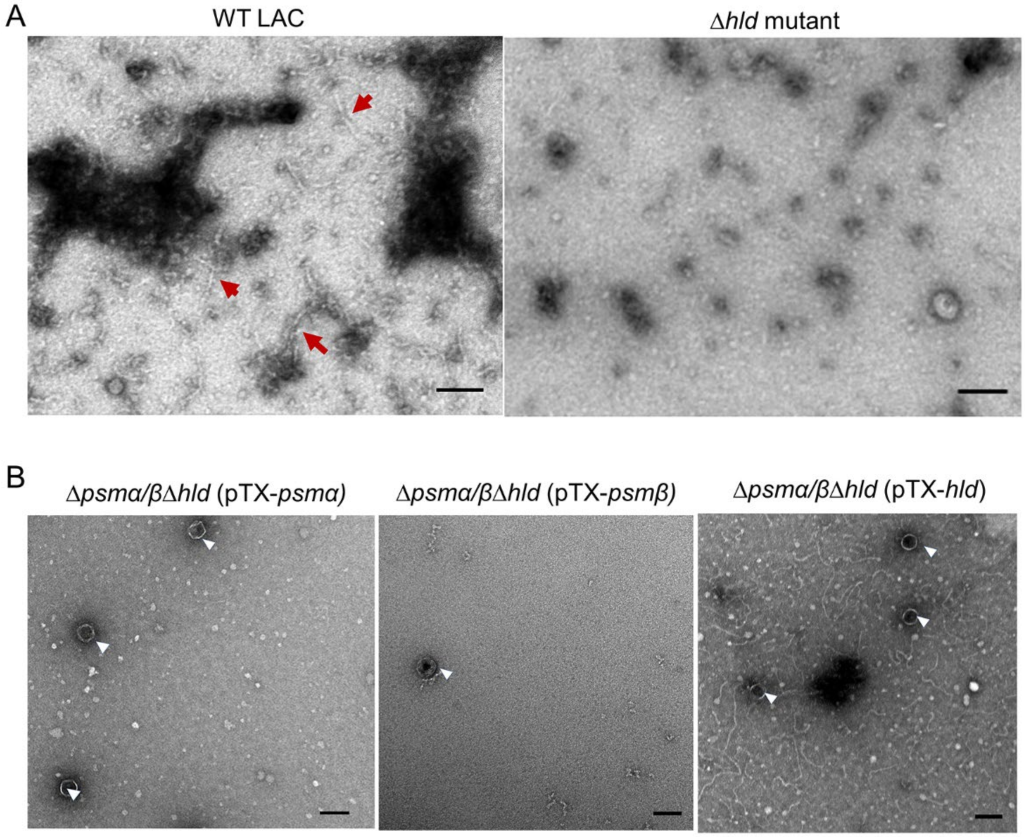

Figure S4. Electron micrographs of samples from WT *S. aureus* and its mutant strains. (A) Culture supernatants of WT LAC and the LACΔ*hld* mutant concentrated by ultrafiltration. Fibrils in the images are marked with red arrowheads. (B) Electron micrographs of MV samples purified from cultures of the Δ*psm*α*/β*Δ*hld* mutant complemented with pTX-*psm*α, pTX-*psm*β, or pTX-*hld* and induced with 0.5% xylose. MVs in the images are marked with white arrowheads. Scale bars, 100 nm.

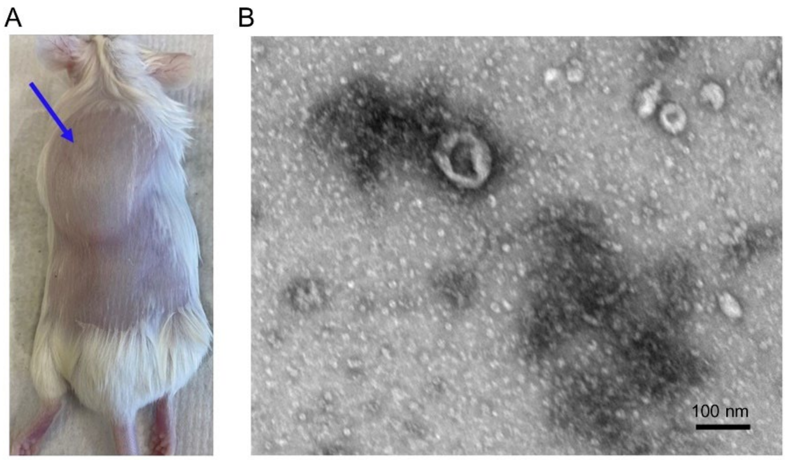

Figure S5. The generation of *S. aureus* MVs in an air pouch infection model. (A) The murine air pouch (arrow) prior to bacterial inoculation. (B) Electron micrograph of crude MVs harvested from air pouch lavage fluids of mice challenged with ~10^8 CFU viable *S. aureus* LAC. Scale bar, 100 nm.

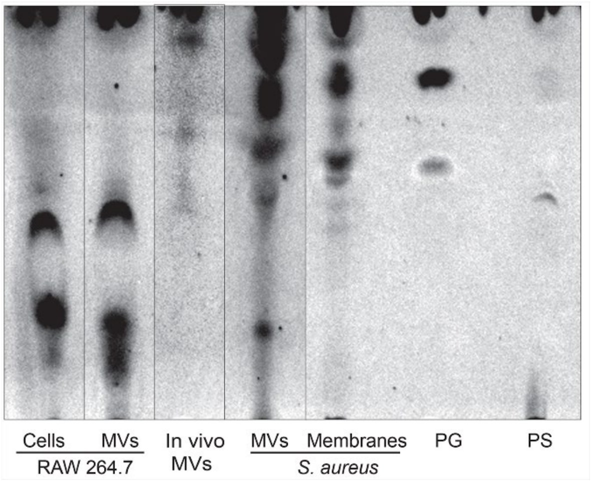

Figure S6. Thin layer chromatogram of lipid extracts of the indicated samples and lipid standards phosphatidylglycerol (PG) and phosphatidylserine (PS).

Reference

1. Wang X, Thompson CD, Weidenmaier C, Lee JC. 2018. Release of *Staphylococcus aureus* extracellular vesicles and their application as a vaccine platform. Nat Commun 9:1379.
