# Supplementary material for: Staphylococcus aureus delta toxin modulates both extracellular membrane vesicle biogenesis and amyloid formation
Source: mBio. 2023 Oct 5;14(5):e01748-23. doi: 10.1128/mbio.01748-23 (PMC10653798; doi:10.1128/mbio.01748-23)
Supplement: Table S1 — Proteins identified in the SDS-PAGE gel band of <10 kDa. [file mbio.01748-23-s0002.docx]

**Table S1.** Proteins identified in the SDS-PAGE gel band of <10 kDa

| Unique peptides | Total peptides | Annotation | Gene | MW (kDa) | Sum intensity | Intensity (%) | Normalized intensity (%)^1^ |
| --- | --- | --- | --- | --- | --- | --- | --- |
| 2 | 36 | Delta-hemolysin | *hld* | 5.01 | 4.30E+09 | 99.980 | 99.988 |
| 3 | 3 | Hypothetical protein | *sausa300_1904* | 6.56 | 4.60E+05 | 0.011 | 0.008 |
| 1 | 1 | 30S ribosomal protein S17 | *rpsQ* | 10.17 | 2.00E+05 | 0.005 | 0.002 |
| 2 | 2 | Foldase protein | *prsA* | 35.62 | 2.80E+04 | 0.001 | 0.000 |
| 1 | 1 | Putative lipoprotein | *sausa*300_0992 | 23.86 | 5.10E+04 | 0.001 | 0.000 |
| 2 | 2 | Pyruvate dehydrogenase E1 component subunit beta | *pdhB* | 35.22 | 4.50E+04 | 0.001 | 0.000 |
| 1 | 2 | Sensor histidine kinase | *lytS* | 64.99 | 2.60E+04 | 0.001 | 0.000 |
| 1 | 1 | Phenol soluble modulin beta peptide 1 | *psmβ1* | 4.49 | 1.80E+04 | 0.000 | 0.000 |
| 2 | 2 | 2-oxo acid dehydrogenase subunit E2 | *pdhC* | 46.35 | 2.10E+04 | 0.000 | 0.000 |

^1^ normalized by molecular weight
