# Supplementary material for: Staphylococcus aureus delta toxin modulates both extracellular membrane vesicle biogenesis and amyloid formation
Source: mBio. 2023 Oct 5;14(5):e01748-23. doi: 10.1128/mbio.01748-23 (PMC10653798; doi:10.1128/mbio.01748-23)
Supplement: Table S2 — S. aureus strains used in this study. [file mbio.01748-23-s0003.docx]

**Table S2.** *S. aureus* strains used in this study

| Strain | Description^a^ | Reference | |
| --- | --- | --- | --- |
| JE2 | USA300 LAC cured of three plasmids | | (1) |
| JE2∆*agr* | JE2 *agr*::*tetM*, Tc^r^ | | (2) |
| JE2∆*atl* | JE2 *atl*::*bursa aurealis*, Em^r^ | | (1) |
| LAC | CA-MRSA, USA300 | | (3) |
| LAC∆*psmα* | *psmα* deletion mutant | | (4) |
| LAC∆*psmβ* | *psmβ* deletion mutant | | (4) |
| LAC∆*psmα/β* | *psmα/psmβ* double mutant | | (5) |
| LAC∆*hld* | *hld* mutant; hld start codon changed from ATG to ATT | | (4) |
| LAC∆*psmα/β∆hld* | *psmα/psmβ/hld* triple mutant | | (5) |
| LAC∆*hld* (pTX-*hld*) | *hld* gene is expressed with inducible vector pTX Tc^r^ | | This study |
| LAC∆*psmα/β∆hld* (pTX*-psmα1-4*) | *psmα1-4* genes are expressed with inducible vector pTX, Tc^r^ | | (6) |
| LAC*∆psmα/β∆hld* (pTX*-psmβ1-2*) | *psmβ1-2* genes are expressed with inducible vector pTX, Tc^r^ | | (6) |
| LAC*∆psmα/β∆hld* (pTX*-hld*) | *hld* gene is expressed with inducible vector pTX, Tc^r^ | | (6) |
| LAC∆*lukAB*∆*hlgACB*∆*lukED*∆*pvl*∆*hla* | Pore-forming toxin mutant lacking *hla* and all leukocidin genes | | (7) |
| MN8 | MSSA, USA200, ST30 | | (8) |
| N315 | HA-MRSA, ST5 | | (9) |
| Sanger 252 | HA-MRSA, ST36 | | (10) |
| MW2 | CA-MRSA, USA400, ST1 | | (11) |
| MW2∆*psmα* | *psmα* deletion mutant | | (4) |
| MW2∆*psmβ* | *psmβ* deletion mutant | | (4) |
| MW2∆*psmα/β* | *psmα/psmβ* double mutant | | (12) |
| MW2∆*hld* | *hld* mutant; hld start codon changed from ATG to ATT | | (4) |
| MW2∆*psmα/β∆hld* | *psmα/psmβ/hld* triple mutant | | (12) |
| NRS483 | CA-MRSA, USA1000, ST59 | BEI Resources | |
| RN4220 | Restriction-deficient mutant of *S. aureus* 8325-4 | (13) | |

^a^Tc, tetracycline; CA-MRSA, community-acquired methicillin resistant *Staphylococcus aureus*; HA-MRSA, hospital-acquired methicillin resistant *Staphylococcus aureus*.

**References**

1. Fey PD, Endres JL, Yajjala VK, Widhelm TJ, Boissy RJ, Bose JL, Bayles KW. 2013. A genetic resource for rapid and comprehensive phenotype screening of nonessential *Staphylococcus aureus* genes. mBio 4:e00537-12.

2. Wang X, Thompson CD, Weidenmaier C, Lee JC. 2018. Release of *Staphylococcus aureus* extracellular vesicles and their application as a vaccine platform. Nat Commun 9:1379.

3. Tenover FC, McDougal LK, Goering RV, Killgore G, Projan SJ, Patel JB, Dunman PM. 2006. Characterization of a strain of community-associated methicillin-resistant *Staphylococcus aureus* widely disseminated in the United States. J Clin Microbiol 44:108-18.

4. Wang R, Braughton KR, Kretschmer D, Bach TH, Queck SY, Li M, Kennedy AD, Dorward DW, Klebanoff SJ, Peschel A, DeLeo FR, Otto M. 2007. Identification of novel cytolytic peptides as key virulence determinants for community-associated MRSA. Nat Med 13:1510-4.

5. Joo HS, Cheung GY, Otto M. 2011. Antimicrobial activity of community-associated methicillin-resistant *Staphylococcus aureus* is caused by phenol-soluble modulin derivatives. J Biol Chem 286:8933-40.

6. Periasamy S, Joo HS, Duong AC, Bach TH, Tan VY, Chatterjee SS, Cheung GY, Otto M. 2012. How *Staphylococcus aureus* biofilms develop their characteristic structure. Proc Natl Acad Sci U S A 109:1281-6.

7. Blake KJ, Baral P, Voisin T, Lubkin A, Pinho-Ribeiro FA, Adams KL, Roberson DP, Ma YC, Otto M, Woolf CJ, Torres VJ, Chiu IM. 2018. *Staphylococcus aureus* produces pain through pore-forming toxins and neuronal TRPV1 that is silenced by QX-314. Nat Commun 9:37.

8. Liu B, Park S, Thompson CD, Li X, Lee JC. 2016. Antibodies to *Staphylococcus aureus* capsular polysaccharides 5 and 8 perform similarly in vitro but are functionally distinct in vivo. Virulence 8:859-874.

9. Kuroda M, Ohta T, Uchiyama I, Baba T, Yuzawa H, Kobayashi I, Cui L, Oguchi A, Aoki K, Nagai Y, Lian J, Ito T, Kanamori M, Matsumaru H, Maruyama A, Murakami H, Hosoyama A, Mizutani-Ui Y, Takahashi NK, Sawano T, Inoue R, Kaito C, Sekimizu K, Hirakawa H, Kuhara S, Goto S, Yabuzaki J, Kanehisa M, Yamashita A, Oshima K, Furuya K, Yoshino C, Shiba T, Hattori M, Ogasawara N, Hayashi H, Hiramatsu K. 2001. Whole genome sequencing of meticillin-resistant *Staphylococcus aureus*. Lancet 357:1225-40.

10. Holden MT, Feil EJ, Lindsay JA, Peacock SJ, Day NP, Enright MC, Foster TJ, Moore CE, Hurst L, Atkin R, Barron A, Bason N, Bentley SD, Chillingworth C, Chillingworth T, Churcher C, Clark L, Corton C, Cronin A, Doggett J, Dowd L, Feltwell T, Hance Z, Harris B, Hauser H, Holroyd S, Jagels K, James KD, Lennard N, Line A, Mayes R, Moule S, Mungall K, Ormond D, Quail MA, Rabbinowitsch E, Rutherford K, Sanders M, Sharp S, Simmonds M, Stevens K, Whitehead S, Barrell BG, Spratt BG, Parkhill J. 2004. Complete genomes of two clinical *Staphylococcus aureus* strains: evidence for the rapid evolution of virulence and drug resistance. Proc Natl Acad Sci U S A 101:9786-91.

11. Baba T, Takeuchi F, Kuroda M, Yuzawa H, Aoki K, Oguchi A, Nagai Y, Iwama N, Asano K, Naimi T, Kuroda H, Cui L, Yamamoto K, Hiramatsu K. 2002. Genome and virulence determinants of high virulence community-acquired MRSA. Lancet 359:1819-27.

12. Cheung GY, Duong AC, Otto M. 2012. Direct and synergistic hemolysis caused by *Staphylococcus* phenol-soluble modulins: implications for diagnosis and pathogenesis. Microbes Infect 14:380-6.

13. Nair D, Memmi G, Hernandez D, Bard J, Beaume M, Gill S, Francois P, Cheung AL. 2011. Whole-genome sequencing of *Staphylococcus aureus* strain RN4220, a key laboratory strain used in virulence research, identifies mutations that affect not only virulence factors but also the fitness of the strain. J Bacteriol 193:2332-5.
